# Comparative Effectiveness of Gemcitabine plus Nab-Paclitaxel and FOLFIRINOX in the First-Line Setting of Metastatic Pancreatic Cancer: A Systematic Review and Meta-Analysis

**DOI:** 10.3390/cancers11040484

**Published:** 2019-04-05

**Authors:** Sara Pusceddu, Michele Ghidini, Martina Torchio, Francesca Corti, Gianluca Tomasello, Monica Niger, Natalie Prinzi, Federico Nichetti, Andrea Coinu, Maria Di Bartolomeo, Mary Cabiddu, Rodolfo Passalacqua, Filippo de Braud, Fausto Petrelli

**Affiliations:** 1 Medical Oncology Department, Fondazione IRCSS Istituto Nazionale Tumori di Milano, 20133 Milan, Italy; sara.pusceddu@istitutotumori.mi.it (S.P.); martina.torchio@istitutotumori.mi.it (M.T.); francesca.corti@istitutotumori.mi.it (F.C.); monica.niger@istitutotumori.mi.it (M.N.); natalie.prinzi@istitutotumori.mi.it (N.P.); federico.nichetti@istitutotumori.mi.it (F.N.); maria.dibartolomeo@istitutotumori.mi.it (M.D.B.); filippo.debraud@istitutotumori.mi.it (F.d.B.); 2 Medical Oncology Department, ASST Cremona, 26100 Cremona, Italy; mghido@hotmail.it (M.G.); g.tomasello@asst-cremona.it (G.T.); r.passalacqua@asst-cremona.it (R.P.); 3 Medical Oncology Department, Ospedale San Francesco, ASSL Nuoro, 08100 Nuoro, Italy; andrea.coinu@atssardegna.it; 4 Medical Oncology and Hemato-Oncology Department, ASST Bergamo Ovest, 24047 Bergamo, Italy; mary_cabiddu@asst-bgovest.it (M.C.); faustopetrelli@gmail.com (F.P.); 5 Department, University of Milan, 20122 Milan, Italy

**Keywords:** pancreatic cancer, FOLFIRINOX, gemcitabine, and nab-paclitaxel

## Abstract

Gemcitabine and nab-paclitaxel (GEM-NAB) and the combination of 5-fluorouracil, oxaliplatin, and irinotecan (FOLFIRINOX) are valid first-line options for advanced or metastatic pancreatic cancer (mPC). However, no randomized trials comparing the two schemes have been performed. This meta-analysis aims to compare GEM-NAB and FOLFIRINOX in terms of safety and effectiveness, taking into account data from real-life studies on mPC. We systematically searched PubMed, EMBASE and Cochrane library up to November 2018 to identify retrospective or cohort studies on mPC comparing GEM-NAB and FOLFIRINOX. We included 16 retrospective studies, including 3813 patients (2123 treated with GEM-NAB and 1690 treated with FOLFIRINOX). Despite a median weighted overall survival (OS) difference in favor of FOLFIRINOX (mean difference: 1.15, 95% confidence interval CI 0.08–2.22, *p* = 0.03), in whole population OS was similar (hazard ratio (HR = 0.99, 95% CI 0.84–1.16; *p* = 0.9). PFS was also not different between the two arms (HR = 0.88, 95% CI 0.71–1.1; *p* = 0.26). The overall response rate was similar (25 vs. 24% with GEM-NAB and FOLFIRINOX). Among grade 3–4 toxicities, neutropenia, febrile neutropenia, and nausea were lower with GEM-NAB, while neurotoxicity and anemia were lower with FOLFIRINOX. In conclusion, despite a numerically longer median OS with FOLFIRINOX as compared to GEM-NAB, the overall risk of death and progression were similar. Their toxicity was different with less nausea, neutropenia, and febrile neutropenia with GEM-NAB, as compared to less neurotoxicity and anemia with FOLFIRINOX. Therefore, analysis of non-randomized “real world” studies to date has not provided evidence of a major benefit of one regimen over the other.

## 1. Introduction

Pancreatic cancer (PC) is the fourth leading cause of cancer-related death in both men and women of the developed countries. Standard treatments include surgery, radiation therapy, chemotherapy, concomitant or sequential chemo-radiotherapy, and targeted therapy. Despite therapeutic improvements, prognosis has remained poor, and for all stages combined, prognosis is dismal with one- and five-year overall survival (OS) rates of only 26% and 5% respectively [1]. About 60% of patients have metastatic pancreatic cancer (mPC) at the time of diagnosis, with a median life expectancy of around one year with current treatments, and in this subgroup the five-year OS rate does not exceed 2% [2]. Current treatment options for mPC involve monotherapy or combined therapies. More recently, the combination of 5-fluorouracil (5-FU), oxaliplatin and irinotecan (FOLFIRINOX), and gemcitabine in association with nab-paclitaxel (GEM-NAB) have yielded significantly improved clinical outcomes compared to gemcitabine monotherapy in pivotal phase III trials as first-line therapy.

In 2011, the ACCORD trial (randomized, phase-3 trial, comparing FOLFIRINOX versus gemcitabine as first-line therapy in ECOG PS 0-1, metastatic pancreatic cancer patients) showed that the FOLFIRINOX regimen was superior to gemcitabine alone in terms of efficacy, with a median OS (mOS) of 11.1 months for FOLFIRINOX versus 6.8 months for gemcitabine (hazard ratio (HR) for death 0.57; *p* < 0.001) [3,4]. The GEM-NAB combination was approved for the first-line treatment of patients with mPC in 2013 based on the results of the MPACT trial. This combination was shown to lengthen survival (mOS of 8.5 months in the GEM-NAB group vs. 6.7 months in the gemcitabine alone group) and delay disease spread [5]. In pivotal trials, FOLFIRINOX showed better survival outcomes numerically than GEM-NAB (mOS of 11.1 vs. 8.5 months, median progression-free survival, mPFS, of 6.4 vs. 5.5 months), with a higher frequency of chemotherapy-related toxicities.

Though the aforementioned randomized controlled trials led to the approval of FOLFIRINOX and GEM-NAB for mPC, there has been no head-to-head comparison of these new treatments in terms of efficacy, cost-effectiveness, and toxicities. In particular, there is no randomized controlled trial comparing GEM-NAB and FOLFIRINOX in the first-line setting. Secondly, cross-trial comparisons are problematic due to differences in trial designs (inclusion criteria more restrictive for the ACCORD trial) and in patients’ baseline characteristics [6,7]. The ACCORD trial was conducted at academic centers in France and enrolled patients aged ≤75 years with an Eastern Cooperative Oncology Group (ECOG) performance status (PS) ≤1. Conversely, the MPACT trial was an international study conducted at academic and community centers and enrolled patients with a Karnofsky prognostic score (KPS) of 70–100 with no upper age restriction.

Given literature heterogeneity, there is a lack of evidence about which regimen is preferable in patients with mPC and which subgroup of patients would benefit more from GEM-NAB or FOLFIRINOX. Usually, FOLFIRINOX is recommended for relatively young patients with a good ECOG PS, while GEM-NAB may be preferred for older patients with a higher ECOG PS [8]. A comprehensive definition of criteria for patient selection for GEM-NAB versus FOLFIRINOX is needed. In this effort, multiple retrospective observational studies comparing effectiveness and safety of GEM-NAB and FOLFIRINOX in “real world” populations were conducted in different countries.

In this article we have performed a meta-analysis on observational retrospective trials and real-life data available for GEM-NAB and FOLFIRINOX as first-line treatment in mPC patients, with the aim of performing a comparative analysis of their safety and effectiveness.

## 2. Results

We found a total of 493 publications in the electronic databases. A total of 16 studies including 3813 patients (2123 and 1690 of whom were treated with GEM-NAB and FOLFIRINOX respectively), were finally selected for inclusion after exclusion of duplicates and other non-pertinent papers [3,6,7,8,9,10,11,12,13,14,15,16,17,18,19,20]. Fourteen were retrospective case series and two were cohort-studies.

Papers were published from 2015 to 2018 (five series were published in abstract form only). All studies except one evaluated patients with metastatic/recurrent or locally advanced inoperable PC. In the Dhir et al. study, among 193 patients with PC, 90% had borderline resectable and 10% resectable disease [10]. The Newcastle-Ottawa scale for the studies ranged from 5 to 8 (mean 6.3), which means that overall the 16 papers were of medium quality. Table 1 details the characteristics of the studies included in the meta-analysis.

### 2.1. Meta-Analysis of OS and PFS

**Figure 1:**
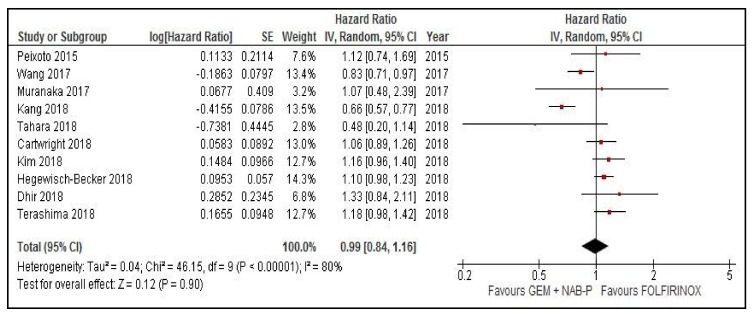
Overall survival comparison between gemcitabine plus nab paclitaxel and FOLFIRINOX. CI: confidence interval, GEM + NAB-P: gemcitabine plus nab paclitaxel, IV: interval variable, SE: standard error.

**Figure 2:**
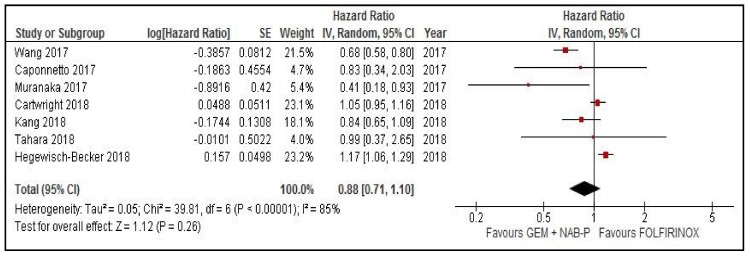
Progression-free survival comparison between gemcitabine plus nab paclitaxel and FOLFIRINOX. CI: confidence interval, GEM + NAB-P: gemcitabine plus nab paclitaxel, IV: interval variable, SE: standard error.

In *n* = 10 studies with data available to obtain HR for OS, the pooled risk of death was similar between arms (HR = 0.99, 95% confidence interval (CI) 0.84–1.16, *p* = 0.9, random effect model, Figure 1). In *n* = 7 studies with data available to compare the risk of progression, the pooled HR was 0.88 (95% CI 0.71–1.1, *p* = 0.26, random effect model, Figure 2). The mean weighted OS difference was 1.15 months (95% CI 0.08–2.22) in favor of FOLFIRINOX (*p* = 0.03). Figure 1 and Figure 2 show OS and PFS comparison between GEM-NAB and FOLFIRINOX, respectively.

### 2.2. Meta-Analysis of Overall Response Rates (ORR)

In *n* = 9 studies, there were data available to calculate the risk of response in the two arms. The pooled odds ratio (OR) for response was 0.93 (95% CI 0.64–1.36, *p* = 0.71, random effect model). The overall response rates (ORR) were 25% and 24% in GEM-NAB and FOLFIRINOX arms, respectively.

### 2.3. Toxicities

Toxicity results are presented in Table 2. Grade 3–4 (G 3–4) adverse events (AEs) with both haematological and non-haematological toxicities were compared in the two arms. Neutropenia and febrile neutropenia were significantly lower in GEM-NAB arms ORs 0.71 (95% CI 0.54–0.92) and 0.45 (95% CI 0.29–0.7) respectively, *p* = 0.01 and <0.001). Conversely, G 3–4 anemia was more frequent with GEM-NAB therapy compared with FOLFIRINOX (ORs 2.5 (95% CI 1.5–4.3), *p* < 0.001). Among non-haematological toxicities, only nausea and neurotoxicity resulted significantly different between the two arms (90% less nausea and 2.5-fold more neurotoxicity with GEM-NAB as compared with FOLFIRINOX). The duration of treatment was similar with three (monthly) cycles for GEM-NAB as compared to 5.4 (biweekly) cycles for FOLFIRINOX. Toxic deaths, dose reduction, or treatment discontinuations were not reported.

### 2.4. Sensitivity Analysis

When we undertook sensitivity analysis with the exclusion of the neoadjuvant study by Dhir et al. [10], in the OS primary analysis, the result did not change (HR = 0.97, 95% CI 0.82–1.15, *p* = 0.71) and the mean OS difference was 1.06 (95% CI 0.01–2.13) in favor of FOLFIRINOX. According to the race of the participants, Asiatic and non-Asiatic studies led to similar results (HR = 0.83, 95% CI 0.53–1.29 and 1.05, 95% CI 0.93–1.19).

### 2.5. Publication Bias

The analysis of OS was found to have no publication bias according to the Egger’s test (*p* = 0.49) and Begg’s test (*p* = 39). These values remained unchanged after using the trim and fill method. To further explore the potential sources of the heterogeneity observed in this analysis, we excluded each study sequentially to determine its effect on the main summary estimate. The HRs ranged from 0.96 to 1.05, and similar results were found for PFS (Begg’s *p* = 0.38 and Egger’s *p* = 0.13). After the one-study removed procedure, PFS HR ranged from 0.8 to 1 in favor of FOLFIRINOX.

## 3. Discussion

Since the advent of GEM-NAB and FOLFIRINOX schedules for the treatment of advanced PC, there has been much debate on the choice of proper first-line treatment. As far as ACCORD and MPACT pivotal studies are concerned, FOLFIRINOX performed better than GEM-NAB for mOS (11.1 vs. 8.5 months, respectively), mPFS (6.4 vs. 5.5 months), and ORR (31.6 vs. 23%) when indirectly compared to GEM alone. However, triplet chemotherapy showed higher toxicity rates, with increased high-grade neutropenia (45.7 vs. 38%), fatigue (23.6 vs. 17%), and diarrhea (12.7 vs. 6%) [4,5].

Up to now, no head-to-head comparison studies on efficacy have been performed and our meta-analysis was aimed at evaluating the retrospective trials and real-life data available on this topic. Considering all these studies of GEM-NAB and FOLFIRINOX treatment as first-line therapy for mPC, no difference in survival outcomes was reported, with HR for PFS of 0.88 (95% CI 0.71–1.1, *p* = 0.26) and HR for OS of 0.99 (95% CI 0.84–1.16, *p* = 0.9). Even after the exclusion of borderline resectable and resectable cases in the sensitivity analysis for OS, results were similar (HR = 0.97, 95% CI 0.82–1.15, *p* = 0.71). The ORRs were almost identical (25 and 24% in GEM-NAB and FOLFIRINOX arms). GEM-NAB was significantly correlated with increased incidence of G 3–4 anemia (HR 2.5, 95% CI 1.5–4.3, *p* < 0.001) and neurotoxicity (HR 2.8, 95% CI 1.4–5.7, *p* = 0.027) with respect to FOLFIRINOX. Fewer cases of G 3–4 neutropenia (HR 0.71, 95% CI 0.54–0.92, *p* = 0.01) and febrile neutropenia (HR 0.45, 95% CI 0.29–0.7, *p* < 0.001) were observed with GEM-NAB as compared to FOLFIRINOX. High-grade nausea was less represented as well in GEM-NAB treated patients (HR 0.11, 95% CI 0.03–0.34, *p* < 0.001). Surprisingly, we found no difference in gastrointestinal toxicities such as diarrhea and vomiting between the two treatments. Moreover, dose reductions and discontinuations did not significantly differ.

Commonly, the use of FOLFIRINOX has been controversial in the first-line treatment setting due to its severe toxicity profile [21]. For this reason, many prospective and retrospective series have reported data with different modified versions of the original triplet schedule, with reduction of at least one of the drugs and/or 5-FU bolus removal [22]. The meta-analysis performed on 11 studies showed a comparative survival benefit between modified FOLFIRINOX and the original schedule, with a reduced rate of G 3–4 AEs for the former, especially in terms of neutropenia (23.1%), febrile neutropenia (4.8%), and fatigue (11.5%) [22]. Recently, attention has shifted towards the use of FOLFIRINOX earlier during the course of the disease, in neoadjuvant and adjuvant settings. Indeed, a recent meta-analysis reported a noticeable role of neoadjuvant FOLFIRINOX in patients with borderline resectable or unresectable disease at diagnosis. After treatment, 43% of patients were resected (95% CI 32.8–53.8), with an R0 resection rate of 39.4% (95% CI 32.4–46.9) [23]. In a recent phase III randomized trial in the adjuvant setting, a modified schedule of FOLFIRINOX was compared to gemcitabine. Triplet chemotherapy led to significant longer survival (mOS 54.4 vs. 35.0 months for gemcitabine, HR 0.64, 95% CI 0.48–0.86, *p* = 0.003). However, G 3–4 AEs occurred more frequently in the combination arm (75.9 vs. 52.9%), with higher incidence of paresthesia (12.7 vs. 0%, *p* < 0.001), sensory peripheral neuropathy (9.3 vs. 0%, *p* < 0.001), diarrhea (18.6 vs. 3.7%, *p* < 0.001), and increased γ-glutamyl transferase levels (18.3 vs. 8.4%, *p* = 0.002) [24]. Modified FOLFIRINOX was tested in second-line treatment after failure of gemcitabine-based treatment obtaining promising results with an acceptable toxicity profile. The ORR was 18.8%, mPFS was 5.8 months (95% CI 3.7–7.9), and mOS was 9.0 months (95% CI 6.4–11.6). High-grade AEs were neutropenia (64.6%) and febrile neutropenia (16.7%) [25].

GEM-NAB has been tested in a non-metastatic setting, as well. While the results of the phase III adjuvant APACT trial are not yet available [26], recent updates on the phase II LAPACT trial of GEM-NAB for locally advanced PC were released. Among 107 enrolled patients, sixteen (15%) underwent surgery after the induction period, ORR was 35% with a mPFS of 10.2 months. The most common G 3–4 AEs were neutropenia (425), anemia (11%), fatigue (10%), and peripheral neuropathy (4%) [27].

Our meta-analysis has clear limitations. Firstly, this is an aggregate analysis of observational retrospective series with arms not adequately balanced in terms of stage and disease burden, performance status, and median age. Secondly, completeness of reporting was also heterogeneous. Many studies did not provide adequate data of follow up and baseline characteristics of patients (biliary stent rate, CA 19.9 and median bilirubin levels). Finally, data of PFS and ORR are local-investigator based with obvious bias, and previous (neo)adjuvant and/or second-line treatments delivered at progression were unknown. This meta-analysis, however, is the first work that indirectly compared, in real-life setting, outcome and toxicity in patients treated with two modern chemotherapy combinations used as first-line therapy in mPC.

As a whole, in the choice of first-line treatment in mPC, many factors are involved. In cases of relapse after adjuvant gemcitabine or gemcitabine-based treatment, relapse-free survival (RFS) duration is crucial in the decision between the two different treatments, with GEM-NAB usable only in the case of patient relapse occurring later than 6 months. The choice of FOLFIRINOX is mainly based on the patient conditions as triplet therapy has a worse toxicity profile compared to GEM-NAB. Moreover, FOLFIRINOX toxicity-related costs, including hospitalization for supportive care, use of pegfilgrastim and anti-emetics, should be taken into consideration in the choice of treatment [28]. GEM-NAB therapy is less expensive than FOLFIRINOX, with costs mainly related to drug acquisition [29]. The recent promising results obtained with FOLFIRINOX both in neoadjuvant and adjuvant may cause a future shift of this treatment from first-line to these settings. On the basis of the increasing number of patients that will receive FOLFIRINOX in locally advanced or resected PC, GEM-NAB could be considered the most suitable first-line treatment in the near future.

## 4. Materials and Methods

This systematic review was based on the recommendations of the guidelines for Meta-analysis of Observational Studies in Epidemiology (MOOSE) [30] and is structured following the PRISMA guidelines.

### 4.1. Search Strategy and Inclusion Criteria

We searched MEDLINE/PubMed, SCOPUS, The Cochrane Library, Web of Science, and EMBASE from first available papers in literature up to November 2018 for potentially eligible studies. For the search we used the terms: pancreatic cancer, gemcitabine, nab-paclitaxel, and *FOLFIRINOX*. We supplemented this search with a manual search of the references for any potential eligible study. We selected retrospective series and prospective nonrandomized or cohort studies of patients with advanced PC. In addition, abstracts published in the main cancer conferences were screened for eligibility, if they reported at least one outcome. The studies compared the effectiveness and/or safety of GEM-NAB and FOLFIRINOX. The inclusion criteria included studies published in English language, patients aged 18 years and older of both sexes, and locally advanced or mPC. Studies were excluded if they were reviews, case reports, studies in animals, were in vitro or phase one studies, investigated other histology types of PC, assessed other concomitant therapies with experimental agents, and included less than 10 patients.

### 4.2. Data Extraction and Quality Assessment

The selection and inclusion of studies were performed in two stages by two independent reviewers (FP and MG). This included the analysis of titles/abstracts followed by the full texts. Disagreements were resolved by a third reviewer (SP). The number of authors, year of publication, characteristics of the patients, median follow up, type of study, as well as effectiveness and safety data were retrieved and incorporated into an appropriate Excel spreadsheet. We used the Newcastle-Ottawa scale to assess the methodological quality of the observational studies [31]. In this scale, each study was assessed in three dimensions, selection of the study groups, comparability of groups, and the calculation of any exposure or outcomes of interest. The total score could be up to nine stars, and studies with a score >6 were considered to be of high quality. The primary outcome measures considered were OS and PFS. The secondary outcome measures were ORR and the occurrence of severe AEs, considering only G ≥ 3.

### 4.3. Statistical Analysis

The results were presented by HRs for continuous variables and expressed as ORs for dichotomous variables, with a 95% CI. To estimate the magnitude of statistical inconsistency, we used the test I^2^ > 50% and a *p* value of <0.10 in the Chi-square test. Values above 50% were considered to have high heterogeneity [32]. An HR < 1 meant a positive result in favor of GEM-NAB combination. A sensitivity analysis was conducted to assess the causes of heterogeneity, by excluding one study at a time (leave-one-out procedure) and observing the changes in the I^2^ and *p* values. The possibility of publication bias was assessed using funnel plots for the outcome. The data from the studies were combined using the random effects model of the Review Manager software, version 5.3.

## 5. Conclusions

The choice between FOLFIRINOX and GEM-NAB in the first-line treatment in mPC is based on many factors, including age, PS, previous adjuvant therapies, toxicities, and treatment costs. With our meta-analysis of nonrandomized “real world” studies, despite a longer median OS with FOLFIRINOX compared to GEM-NAB, we did not register a major benefit of one regimen over the other in terms of overall risk of death and progression.

## Figures and Tables

**Table 1:** Characteristics of included studies gemcitabine and nab-paclitaxel (GEM-NAB) vs. 5-fluorouracil, oxaliplatin, and irinotecan (FOLFIRINOX).

Author/Year	N° pts	Country	Type of Study	Follow up (Months)	PS 0–1% GN/F	Median Age GN/F (Years)	Stage (LAD/Metastatic) GN vs. F	Abnormal Bilirubin % GN vs. F	Liver Metastases % GN vs. F	Location(Head) GN vs. F	CA 19.9 (Median) GN vs. F	Biliary Stent % GN vs. F
Braiteh 2017 [9]	202	USA	Retrospective	-	82 vs. 82	69/61.5	0/88 vs. 0/89	-	64 vs. 73	-	48 vs. 37	-
Dhir 2018 [10]	193	USA	Retrospective	27.5	-	69/63	59.2 vs. 79.4/0 vs. 0	-	-	-	235 vs 243	-
Cartwright 2018 [6]	314	USA	Retrospective	-	76.8/90.5	68/61	0/100 vs. 0/100	-	33.1 vs. 33.5	-	-	-
Kang 2018 [8]	308	Korea	Retrospective	19.6	96.6/99. 4	62/60	0/100 vs. 0/100	-	61.1 vs. 67.9	32.2 vs. 39.7	443 vs. 576	-
Hegewisch-Becker 2018 [11]	773	Germany	Retrospective	-	89.6 vs. 95.8	71/60	-/89 vs. 90	9.8 vs. 7.4	-	52.4 vs. 52.5	-	-
Kim 2018 [12]	654	USA	Retrospective	9	70.3 vs. 91.5	64.59/59.03	0/100 vs. 100	-	75.34 vs. 75.73	50.5 vs. 51.3	-	-
Muranaka 2017 [13]	38	Japan	Retrospective	11.9–8.3 (F-GN)	100 vs. 100	66.5/63	13.6 vs. 43.8/86.4 vs. 56.2	-	-	-	305.6 vs. 128.7	9.1 vs. 18.8
Peixoto 2015 [7]	331	Canada	Retrospective	-	-	-	-	-	-	-	-	-
Tahara 2018 [14]	27	Japan	Retrospective	-	100/100	63 vs. 62	-	0 vs. 0	26.6 vs. 16.6	66.6 vs. 50	-	-
Terashima 2018 [15]	67	Japan	Retrospective	5.6	- vs. 85.1	-	-	-	-	-	-	-
Wang 2017 [16]	179	Canada	Retrospective	8.5	59/93	68/60	-/75.9 vs. 59.8	10 vs. 10	34.8 vs. 30.1	48.3 vs. 67.4	1228 vs. 415	20.7 vs. 32.6
Caponnetto 2017 [17]	43	Italy	Retrospective	-	100/100	-	0/100 vs. 0/100	-	-	-	-	-
Cho 2018 [18]	167	Korea	Cohort study	7.9	-	65/54	0/100 vs. 0/100	-	-	-	-	-
Kasi 2017 [19]	154	US	Retrospective	-	83/90	63/61	30/70 vs. 48/52	-	-	55 vs. 63	-	20/41
Schmidt 2016 [3]	56	US	Retrospective	-	-	-	100/100	-	-	-	-	-
Yamamoto 2017 [20]	207	Japan	Retrospective	-	-	-	-	-	-	-	-	-

GN: Gemcitabine—Nab-paclitaxel; F: FOLFIRINOX; LAD: locally advanced disease; N°: number; pts: patients; PS: performance status

**Table 2:** Toxicity results for FOLFIRINOX compared to gemcitabine plus nab-paclitaxel arms.

Toxicity G 3–4 GEM-NAB vs. FOLFIRINOX	N° Studies	Pooled Effect (OR, 95% CI)	I^2^ (%)	Analysis Model	*p*
Hematologic					
Neutropenia	8	**0.71 (0.54–0.92)**	0	Fixed	0.01 *
FN	5	**0.45 (0.29–0.7)**	0	Fixed	<0.001 *
Anemia	7	**2.5 (1.5–4.3)**	0	Fixed	<0.001 *
Thrombocytopenia	6	0.89 (0.37–2.1)	0	Random	0.79
Non-hematologic					
Stomatitis	NA				
Diarrhea	5	0.27 (0.03–2.2)	0	Fixed	0.23
Nausea	2	**0.11 (0.03–0.34)**	0	Fixed	<0.001 *
Vomiting	2	0.76 (0.03–19.5)	0	Fixed	0.87
Anorexia	2	0.41 (0.05–3.2)	0	Fixed	0.4
Fatigue	2	1 (0.2–4.9)	0	Fixed	1
Neurotoxicity	5	**2.8 (1.4–5.7)**	31.9	Fixed	0.027 *
Infections/sepsis	NA				
ALT increase	NA°				
AST increase	NA°				
Toxic deaths	NA°				
Thrombosis	NA				
Dose reduction	4	0.81 (0.46–1.4)	69.9	Random	0.45
Discontinuations	2	1.26 (0.23–6.87)	79.3	Random	0.78
Median cycle	3	3 vs. 5.4			

°, in 1 study the rate was 0% in both arms, ALT: alanine aminotransferase, AST: aspartate aminotransferase, CI: confidence interval, FN: febrile neutropenia, G: grade, GEM-NAB: gemcitabine +plus nab-paclitaxel, N°: number, NA: not available, OR: odds ratio, *: statistically significant *p*-values.
